# Rising to the Challenge of Early Screening in Primary Health Care Through the Web Italian Network for Autism Spectrum Disorder (Win4ASD) in the Pediatric Population: Retrospective Observational Study

**DOI:** 10.2196/74302

**Published:** 2026-02-24

**Authors:** Noemi Buo, Eleonora Rosi, Silvia Busti Ceccarelli, Mariarosa Ferrario, Valerio Maiorca, Erika Morandi, Nicole Viganó, Ivan Limosani, Laura Falcone, Massimo Molteni, Paola Colombo

**Affiliations:** 1 Child Psychopathology Unit-Smart Lab Scientific Institute, Istituto di Ricovero e Cura a Carattere Scientifico (IRCCS) Eugenio Medea Bosisio Parini, Lecco Italy; 2 Azienda Socio Sanitaria Territoriale della Valle Olona - Dipartimento di salute mentale e delle dipendenze Gallarate, Varese Italy; 3 Direzione Generale Welfare, Lombardy Region Milan Italy

**Keywords:** screening Checklist for Autism in Toddlers, CHAT, online screening, tele–mental health, primary care, early detection, autism spectrum disorder, health care policy, surveillance system

## Abstract

**Background:**

Early identification of autism spectrum disorder (ASD) and neurodevelopmental disorders is a key element in the ability to intervene early and improve children’s outcomes. The American Academy of Pediatrics recommends routine autism screening for children aged 18 to 24 months. Telemedicine (health care services delivered remotely using digital technologies) is a valuable tool in supporting this objective. As highlighted in the literature, telemedicine increases the availability of treatment, reduces diagnostic waiting times, and supports the monitoring of neurodevelopmental disorders. The Web Italian Network for Autism Spectrum Disorder (Win4ASD) platform is a telemedicine-based screening tool designed to detect ASD early within the pediatric health care system in Italy.

**Objective:**

This study investigated the integration of the Win4ASD platform into the Italian National Health Service, providing a detailed account of its operational features and use patterns among family pediatricians. It further examined the platform’s contribution to reinforcing preventive health care strategies and enhancing coordination across child health services. Finally, through a retrospective observational analysis, this study evaluated the clinical effectiveness of Win4ASD in facilitating the early detection of ASD and improving screening outcomes.

**Methods:**

The Win4ASD platform, which is active in the Lombardy region, helps family pediatricians screen toddlers for autism at an early age (18-24 months) using the Checklist for Autism in Toddlers. Furthermore, children identified as at risk are referred for further diagnostic evaluation to the local specialized autism unit—a mental health specialist team comprising child neuropsychiatrists, psychologists, and developmental therapists. This platform, which created an integrated network for early identification of autism, ensures rapid access to diagnostic and therapeutic services. This study analyzed data collected from the platform between January 2022 and September 2024 to assess family pediatricians’ commitment and effectiveness in identifying children at risk of autism using descriptive analyses performed in SPSS.

**Results:**

The first key finding was the commitment of family pediatricians from the Regional Public Health Services in Lombardy to use the platform. In total, 72.5% (909/1253) of all family pediatricians working for the Regional Public Health Services actively used the platform. The platform enabled the screening of 58,419 infants from the general population in Lombardy, of whom 596 (1%) were identified as “at risk” and referred for fast-track diagnostic evaluation, which is consistent with global prevalence rates. In addition, diagnostic outcomes from a subsample are presented, showing the effectiveness of the platform in facilitating the early diagnosis of neurodevelopmental disorders.

**Conclusions:**

Early identification of risk and timely intervention can prevent the progressive development of behavioral atypicalities, have a significant impact on the quality of life of the child and their family, and allow for a reduction in health care costs and a consequent improvement in the organization of the health care system.

## Introduction

### Prevention of Neurodevelopmental Disorders and Telemedicine

Telemedicine refers to the full range of health care services delivered through innovative digital technologies whereby the health professional and the patient are not in the same location [1,2]. Recently, telemedicine interventions have gained significant interest and expanded into many areas of medicine, including mental health, both before and after the COVID-19 pandemic [3-5]. The pandemic first increased the demand for effective health support, and the growing need for easy-to-access remote services has substantially boosted the use of telehealth since then [5,6]. According to recent reviews, telehealth can potentially increase treatment availability; reduce diagnostic waiting times; and aid in the monitoring of mental disorders, particularly neurodevelopmental disorders [5,7].

Neurodevelopmental disorders, such as autism spectrum disorder (ASD) and attention-deficit/hyperactivity disorder, typically begin in childhood and may persist as chronic conditions throughout life [8]. Neurodevelopmental conditions account for the highest estimated prevalence in children aged <15 years worldwide, ranging from 4.1% (<5 years) to 7% (10-14 years) [9]. The World Health Organization emphasizes that identifying children at risk of neurodevelopmental disorders is a critical first step to establishing a strong relationship between parents and health care professionals to ensure early intervention [10]. Early intervention aims to prevent or minimize motor, cognitive, and emotional impairments in young children disadvantaged by biological or environmental risk factors [11].

In summary, the high prevalence of neurodevelopmental disorders and the need for early intervention make prevention and screening more crucial than ever. Telemedicine can be an effective tool in identifying these disorders at an early stage.

### The Health Care System in Italy

The Italian National Health Service (NHS) is a public, regionally based system organized at the national, regional, and local levels [12] and provides free primary care, inpatient care, and health screenings for all residents. Public health care is financed primarily through national and regional taxes (97%) and patient copayments. Private health insurance plays a limited role in Italy’s health coverage system [13].

The pediatric health care system in Italy is part of the NHS and is organized in the traditional 3 levels of intervention: primary care (first access), secondary care (hospital care), and tertiary care (specialized hospital care) [14]. Children are required to register with a family pediatrician until the age of 6 years, with the option to remain under pediatric or general practitioner care until the age of 14 years. At 14 years, patients transition to general practitioners, although extensions with pediatricians may be granted for chronic or disabling conditions. Both family pediatricians and general practitioners serve as primary care providers within the system [15].

Children’s development is monitored by family pediatricians at predetermined time points, totaling 10 visits from birth to the age of 13 years, during well-child checkups. Additionally, family pediatricians are responsible for implementing specific health promotion initiatives, providing parents with valuable information to assess their children’s growth and health status, diagnosing and treating illnesses, prescribing medications and diagnostic tests when necessary, referring patients to specialized facilities, and collaborating with other health care professionals.

### Early Prevention System for ASD

ASD is a neurodevelopmental disorder characterized by social and communication impairments and restricted or repetitive behaviors [8,16]. It has a median global prevalence of 65 in 10,000 and 1 in 31 children according to Centers for Disease Control and Prevention data [17-19]. Specifically, a recent systematic review reported a prevalence of ASD between 1.70% and 1.85% in American children aged 4 and 8 years, respectively, whereas the prevalence in Europe ranges between 0.38% and 1.55% [20]. In Italy, a very recent nationwide population study estimated a prevalence of 13.4 (CI 11.3-16.0) per 1000 children aged 7 to 9 years, with a male-to-female ratio of 4.4:1 [21].

The presentation and onset patterns of ASD are variable. In many cases, the core diagnostic features of autism can be identified during toddlerhood [22]. Interventions are more beneficial when delivered earlier in life [23,24], and the American Academy of Pediatrics recommends routine autism screening at 18 to 24 months [16]. Furthermore, over the past 12 years, the American Academy of Pediatrics has reported an increase in the prevalence rates of ASD in children, an expanded understanding of potential risk factors, improved awareness of co-occurring medical conditions and genetic contributions to the etiology, and substantial growth in research supporting evidence-based interventions [16]. Recent studies have emphasized the importance of early ASD detection through screening [7,23,24]. Standardized, digital, high-fidelity ASD screening during pediatric well-child visits facilitates the identification of children at higher risk of autism at a younger age, including those with more subtle clinical manifestations [7,25-27].

In Italy, guidelines on the diagnosis and treatment of ASD in children and adolescents have been approved and published [28,29], emphasizing the importance of early diagnosis.

Given the importance of telemedicine in the prevention and active surveillance of mental health, a screening system, the Web Italian Network for Autism Spectrum Disorder (Win4ASD) platform [30,31], has been developed in Lombardy, a highly populated Italian region with 10,012,054 inhabitants [32]. It provides early identification of individuals at risk of neurodevelopmental disorders using the Checklist for Autism in Toddlers (CHAT) [33]. Owing to computerized scoring, children identified as being at risk are immediately referred by the platform to the local NHS child psychiatric services for a fast-track diagnostic evaluation. The platform aims to create an integrated, interdisciplinary network, enabling early identification of ASD risk; facilitating rapid activation of diagnostic pathways; and ensuring care, habilitation, and rehabilitation based on priorities and intensities determined by the child’s age group and functioning profile.

On the basis of these premises, this paper aims to illustrate how the Win4ASD platform has become part of the public NHS and its potential as a preventive medicine model for the general population through a retrospective observational study.

The primary objective of this study was to illustrate the integration of the Win4ASD platform within the NHS, detailing its functionalities and analyzing the associated screening pathways. Furthermore, this study aimed to evaluate the platform’s contribution to the reorganization of public health care services, the strengthening of preventive strategies, and the enhancement of coordination among health care professionals and facilities. Finally, the clinical effectiveness of the platform was assessed in a subsample of users through a retrospective observational study with the objective of determining how the use of Win4ASD may facilitate the early diagnosis of ASD and improve screening outcomes.

## Methods

### Overview

The Win4ASD platform enables early screening for autism in toddlers (from 18 months; –2 months to +2 months) by filling out the CHAT [33].

The family pediatrician accesses the platform to register the patient and record project consent and then administers the screening. The CHAT screening test involves the administration of 14 items: 9 questions directed to the parents and 5 direct observations of the child’s behavior, which are assessed in person by the pediatrician, who provides the corresponding responses for these observational items and enters them into the platform. Upon completion, the platform provides automated scoring in real time; stores the results; and, based on the screening outcome, recommends appropriate follow-up actions for the family pediatrician to undertake.

If the screening outcome is “generic” or “medium risk,” the assessment can be repeated after a month. In the case of a “high risk” outcome at the first screening and a “generic risk,” “medium risk,” or “high risk” outcome at the repeated screening, the Win4ASD platform allows for the patient’s referral to fast-track diagnostic evaluation. This customized platform is designed to connect primary care (family pediatricians) and mental health specialist services in safe ways. Specifically, 8 health care protection agencies in Lombardy provided a list of all NHS pediatricians, who then received invitations to use the platform. Within the platform, each family pediatrician has the option to refer high-risk children to the local specialized autism unit (SAU), a multidisciplinary and specialized team focused on the diagnosis and treatment of ASD, based on the gold standard. Specifically, each diagnostic assessment includes standardized procedures—comprising parent or caregiver interviews, direct child observations, and administration of validated diagnostic instruments (eg, the Autism Diagnostic Observation Schedule, Second Edition [ADOS-2]; Autism Diagnostic Interview–Revised [ADI-R]; Vineland Adaptive Behavior Scales, Second Edition; Leiter International Performance Scale; Griffiths Scales of Child Development, Third Edition; Psychoeducational Profile, Third Edition; Protocol for a Preventive Assessment of the Motor Coordination Disorder; *Primo Vocabolario del Bambino*; and Adaptive Behavior Assessment System–II)—conducted by clinical psychologists and child psychiatrists. The final diagnosis is formulated by the child psychiatrist, who integrates all clinical and psychometric data to develop the child’s functional profile and therapeutic plan. While the diagnostic process is standardized across local units, individual teams may incorporate additional assessments according to clinical needs [34-43].

The different services communicate through the platform bulletin board, where pediatricians and mental health specialists can write observations and attach documents or reports. In addition, e-learning training courses and information on how to use the platform for family pediatricians are visible and easily accessible from the platform dashboard. The dashboard aggregates key information for real-time monitoring of screening data, patient data, and the activity trends of family pediatricians. The platform is described in detail in the study by Colombo et al [31]. For this study, we considered family pediatricians’ activities and screenings performed within the platform from January 1, 2022, to September 30, 2024. The effectiveness of the screening process was studied further by analyzing data from a subsample for which outcome data were available.

The platform is now being used throughout the Lombardy region following the approval of the Autism Operational Plan (regional deliberations XI/5415 of October 25, 2021, and XII/3686 of December 20, 2024) [34].

### Ethical Considerations

This study was conducted in accordance with the ethical principles for research involving human participants and with the guidelines of the Declaration of Helsinki. It was reviewed and approved by the Eugenio Medea Scientific Institute Ethics and Committee (protocol code 352; September 22, 2016). Informed consent was obtained from the caregivers of all participating children before data collection through pediatric clinical practice. All data used in this research were pseudonymized to ensure participants’ privacy and confidentiality. No financial or material compensation was provided for participation in the study. The dataset analyzed does not contain any information that could allow for the identification of individual participants.

### Materials

#### Win4ASD Platform

We used the input from the Win4ASD platform, representing “performance data” together with data resulting from screening. Specifically, we calculated the number of family pediatricians per participating district in the Lombardy region and subsequently categorized them into 3 groups: those who were active and regularly used the platform, those who had never accessed it, and those who had logged in but did not engage with it.

#### Screening Tool

The screening tool is the Italian version of the CHAT [33,44]—a checklist for detecting children aged 18 months (–2 months to +2 months) who are at risk of autism. It was validated in England with a population of 16,000 children and was designed to be administered by primary health care services. The checklist consists of 2 sections: section A comprises 9 questions for parents asked by the family pediatricians, while section B comprises 5 items completed by the pediatrician based on direct observation of the child’s behavior. All items are rated as “yes” or “no.” This standardized screening tool is available to family pediatricians through the web application. It takes 5 to 10 minutes to administer and requires the family pediatricians’ direct observation and involvement in the entire process, thereby increasing their compliance. The screening result can be “high risk” (failure of all critical items), “medium risk” (failure of a few critical items), “generic risk” (a risk of other neurodevelopmental disorders; failure of a few noncritical items), or “no risk.” If the outcome of the first evaluation is “medium risk” or “generic risk,” the family pediatrician has the option of repeating the assessment after 1 month (second evaluation). If the outcome is “high risk” either at the first or repeated screening or “medium risk” or “generic risk” at the repeated evaluation, the family pediatrician refers the patient for fast-track diagnostic evaluation through the platform.

### Diagnostic Outcome

The diagnostic results are available for a subsample derived from a specific SAU in a regional district (Valle Olona). The diagnostic procedures described above (eg, clinical interviews and observations of the child) were carried out, and in particular, the 2 gold standard tools for autism were used: the ADOS-2 [35] and ADI-R [45]. The ADOS-2 is a semistructured, standardized assessment of autism symptomatology across communication, reciprocal social interaction abilities, play, and restricted and repetitive behaviors and yields diagnostic classifications or concern classification scores. The ADI-R is an investigator-based semistructured diagnostic interview that was designed to provide a developmental history framework for a lifetime differential diagnosis of pervasive developmental disorders and information about current functioning (over the previous 3 months) for individuals with a mental age of ≥2 years from early childhood to adult life. It is usually conducted with the parents or main carer of the child.

### Statistical Analysis

Analyses were performed both on the total Win4ASD platform sample and on a specific subsample from the Valle Olona SAU. Descriptive statistics were used to summarize demographic characteristics and screening outcomes. Continuous variables were expressed as means and SDs, and categorical variables were expressed as absolute and relative frequencies. The 95% CIs were calculated for the main outcome proportions to assess the precision of estimates. Additionally, comparisons between male and female participants were conducted to explore potential Sex-related differences in screening outcomes. All statistical analyses were performed using SPSS (version 26; IBM Corp) [46], and a 2-sided significance level of .05 was considered.

### Data Exclusion

We excluded the CHATs completed outside the age range of 16 to 22 months from the overall sample. In addition, as mentioned previously, we focused on a time frame that excluded all CHATs completed before January 2022 and after September 30, 2024. Of the total sample, 16 participants were excluded from the repeat assessment because they repeated the CHAT despite being classified as “no risk” at the first assessment.

## Results

### Win4ASD Platform Data

#### Pediatricians’ Commitment

A total of 1253 family pediatricians were registered on the platform during the indicated time frame, of whom 909 (72.5%) were active and regularly used the platform, 188 (15%) had never logged on, and 163 (13%) had logged on but were not using the platform. The average number of screening CHATs performed by family pediatricians was 70.5 (SD 53.9; range 1-317). Through the platform’s interactive dashboard, all information is presented and synthesized simply and intuitively.

**Figure 1:**
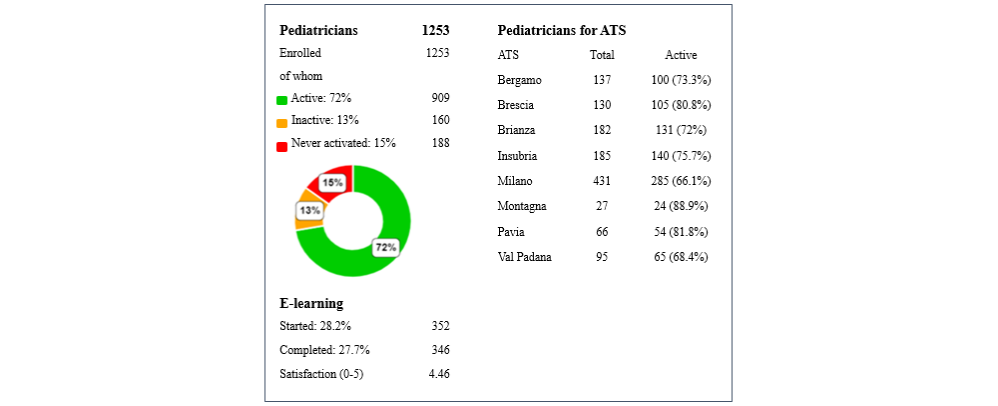
Family pediatricians’ commitment to the web platform (total number of pediatricians, pediatricians who have taken e-learning courses, pediatricians divided by local ATS department). ATS: Italian name for the 8 local health authorities.

**Figure 2:**
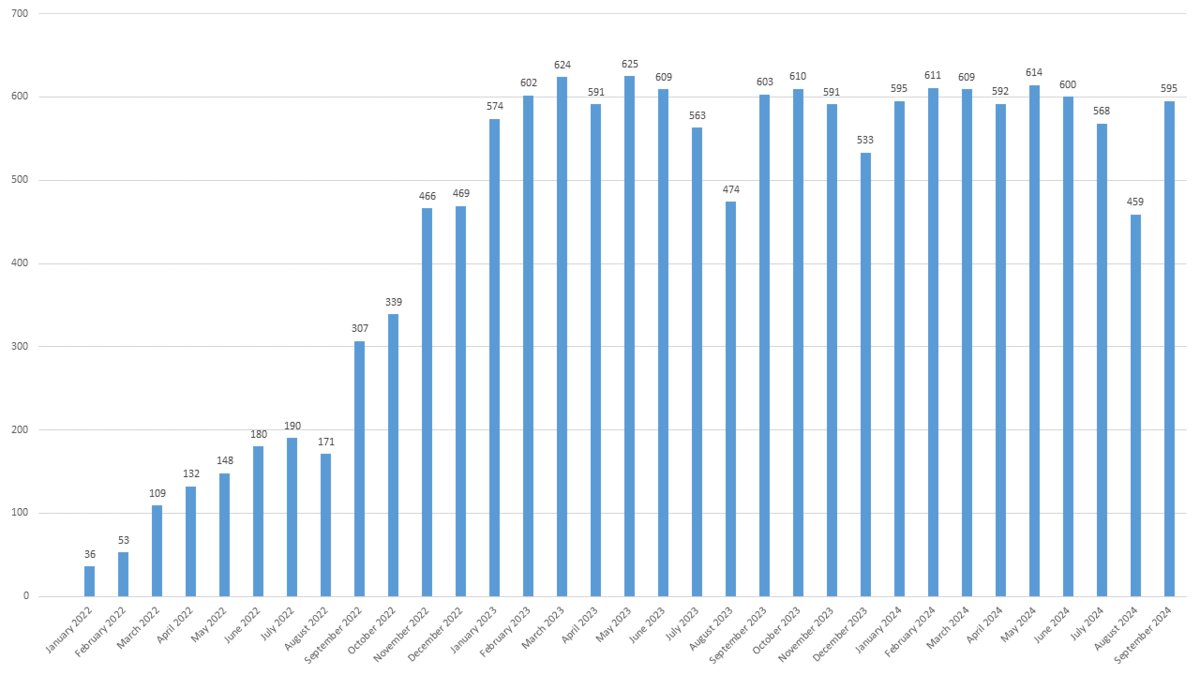
Family pediatricians’ commitment over time: the x-axis shows the different months from January 2022 to September 2024, whereas the y-axis represents the number of pediatricians who registered on the platform and actively used it.

The data shown in Figure 1 illustrate the pediatricians’ use of the platform across different regional districts and their inclusion in the e-learning program (n=346). The bar chart in Figure 2 provides information about family pediatricians’ engagement over time.

#### Early Screening System

A total of 58,419 toddlers from the general population in the Lombardy region were screened during the 16- or 18-month well-child checkup visit (n=29,980, 51.3% male and n=28,439, 48.7% female; mean age 17.32, SD 1.35; range 16-22 months).

**Figure 3:**
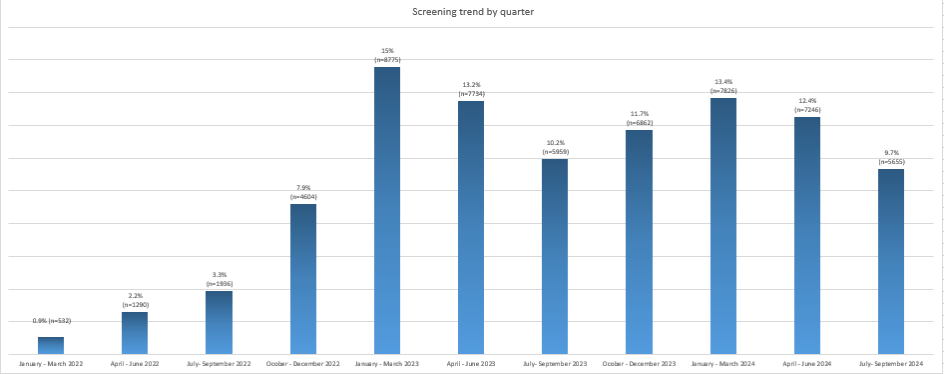
Number of screenings by family pediatricians from January 2022 to September 2024 shown by quarterly trends.

From January 2022 to September 2024, the number of screenings gradually increased from 532 in the first quarter of 2022 to 5666 in the third quarter of 2024. The largest number of screenings was performed by family pediatricians in the first quarter of 2023, reaching 8775 CHATs (Figure 3).

**Figure 4:**
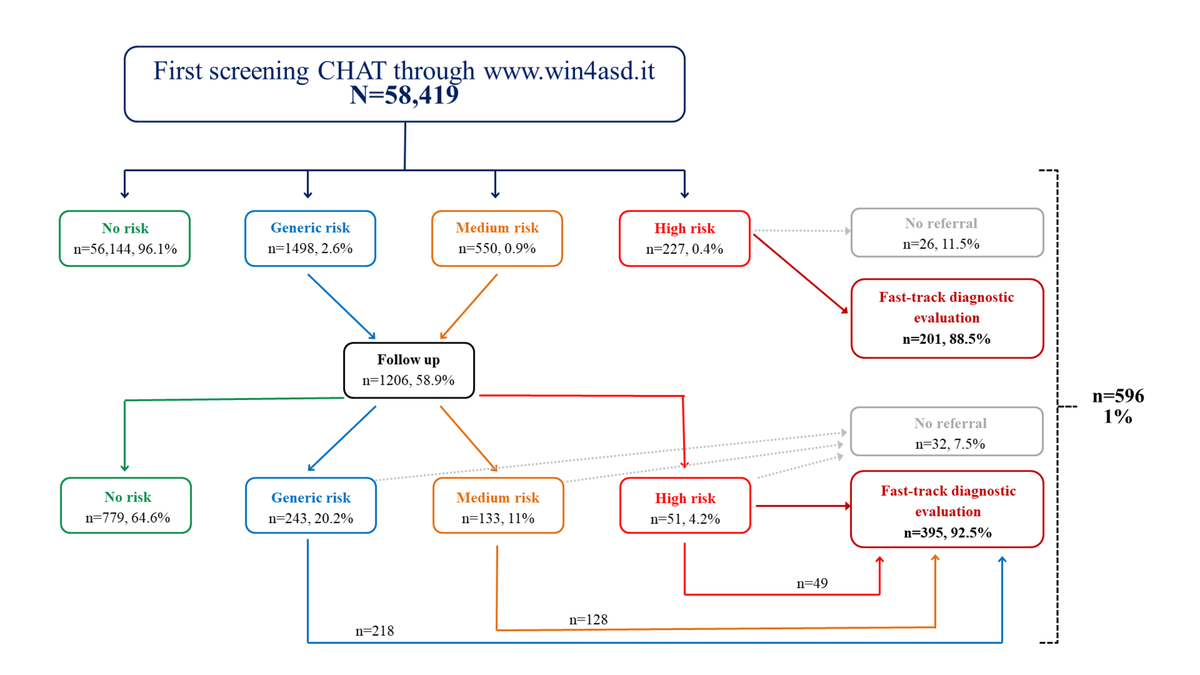
Screening outcomes: flowchart of screenings conducted by family pediatricians from January 2022 to September 2024. CHAT: Checklist for Autism in Toddlers.

A flowchart (Figure 4) shows the outcomes for patients with completed first and repeated screening CHATs, as well as referrals for a further diagnostic investigation.

On the basis of the results, 96.1% (56,144/58,419) of the children were at “no risk” of a neurodevelopmental disorder at first screening, 2.6% (1498/58,419) had a “generic risk,” 0.9% (550/58,419) were at “medium risk,” and 0.4% (227/58,419) were at “high risk.” Table 1 details the proportions of each screening outcome with corresponding 95% CIs in the overall screened population. A total of 0.3% (201/58,419) of the children screened in total (201/227, 88.5% of “high risk” children) were referred for fast-track diagnostic evaluation to the SAU. The detailed screening outcomes with a comparison of demographic variables is shown in Table 2.

**Table 1:** Proportions of each screening outcome with corresponding 95% CIs in the overall screened population (N=58,419).

Screening outcome	Participants, n (%; 95% CI)
No risk	56,144 (96.1; 95.95-96.26)
Generic risk	1498 (2.6; 2.44-2.69)
Medium risk	550 (0.9; 0.86-1.02)
High risk	227 (0.4; 0.34-0.44)

**Table 2:** Comparison of demographic and screening characteristics between male and female participants (N=58,419).

Variable		Male (n=29,980)	Female (n=28,439)	*P* value	
Age at screening (months), mean (SD)		17.33 (1.36)	17.31 (1.33)	.03	
**Screening outcome, n (%)**					<.001
	No risk	28,552 (95.2)	27,592 (97)		
	Generic risk	917 (3.1)	581 (2)		
	Medium risk	581 (1.9)	192 (0.7)		
	High risk	153 (0.5)	74 (0.3)		

In total, 11.5% (26/227) of the participants identified as “high risk” by the CHAT but who were not referred to the SAU were considered out of sample for several reasons. Of these 26 participants, 2 (7.7%) were excluded as they had moved away and were no longer receiving care at the appropriate SAU. In total, 23.1% (6/26) of the participants were already receiving care at other facilities. Regarding the remaining 69.2% (18/26), no information was available; lack of data may have been due to a family pediatrician’s failure to use the platform correctly or a shared decision with the family not to proceed with referral of the child to the SAU.

Participants found to be at “generic risk” and “medium risk” (2048/58,419, 3.5%) repeated the screening after a month, as outlined in the protocol; among these 2048 participants, 1206 (58.9%) follow-ups were made.

Of the 1206 patients for whom screening was repeated (mean age at repeat screening 18.41 months), 779 (64.6%) were classified as “no risk,” and 427 (35.4%) were identified as at risk. Of these 1206 patients, 243 (20.1%) were at generic risk, 133 (11%) were at medium risk, and 51 (4.2%) were at high risk. In total, 92.5% (395/427) of the patients at risk (395/58,419, 0.7% of the total sample) were referred for fast-track diagnostic evaluation to the SAU. In total, 1% (596/58,419) of the children in the total sample (399/596, 66.9% male; 197/596, 33.1% female) were referred for fast-track evaluation to the SAU.

In total, 7.5% (32/427) of the patients identified as “at risk” at the repeated assessment but who were not referred to the SAU were considered out of sample. No information was available. Again, the lack of data may have been due to a pediatrician’s failure to use the platform or a shared decision with the family not to proceed with referral.

### Assessing the Effectiveness of Screening

The performance of the CHAT screening test was evaluated by comparing the actual test results with the patients’ true diagnostic status, as determined through a gold standard assessment. Among the children who underwent screening, diagnostic investigation outcomes were available for a subsample drawn from the Valle Olona SAU. This selection was driven by clinical and organizational considerations and, therefore, represents a convenience sampling [47]. Although this sampling approach may limit generalizability, a comparison between the Valle Olona SAU cohort and the rest of the population was performed using 2-tailed *t* tests and chi-square tests (Table 3). Given the large overall sample size (N=58,419), even minimal absolute differences reached statistical significance (eg, mean age 17.31, SD 1.34 months vs mean age 17.46, SD 1.42 months in the other SAUs and the Valle Olona SAU, respectively; *P*<.001).

**Table 3:** Comparison of demographic and screening characteristics among the overall screened population, the population at other centers, and the Valle Olona specialized autism unit (SAU; N=58,419).

Variable		Total sample	Valle Olona SAU (n=3599)	Other SAUs (n=54,820)	*P* value	
Age at screening (months), mean (SD)		17.32 (1.35)	17.46 (1.42)	17.31 (1.34)	<.001	
**Sex, n (%)**						.62
	Male	29,980 (51.3)	1832 (50.9)	28,148 (51.3)		
	Female	28,439 (48.7)	1767 (49.1)	26,672 (48.7)		
**Screening outcome, n (%)**						.02
	No risk	56,144 (96.1)	3427 (95.2)	52,717 (96.2)		
	Generic risk	1498 (2.6)	107 (3)	1391 (2.5)		
	Medium risk	550 (0.9)	47 (1.3)	503 (0.9)		
	High risk	227 (0.4)	18 (0.5)	209 (0.4)		

**Figure 5:**
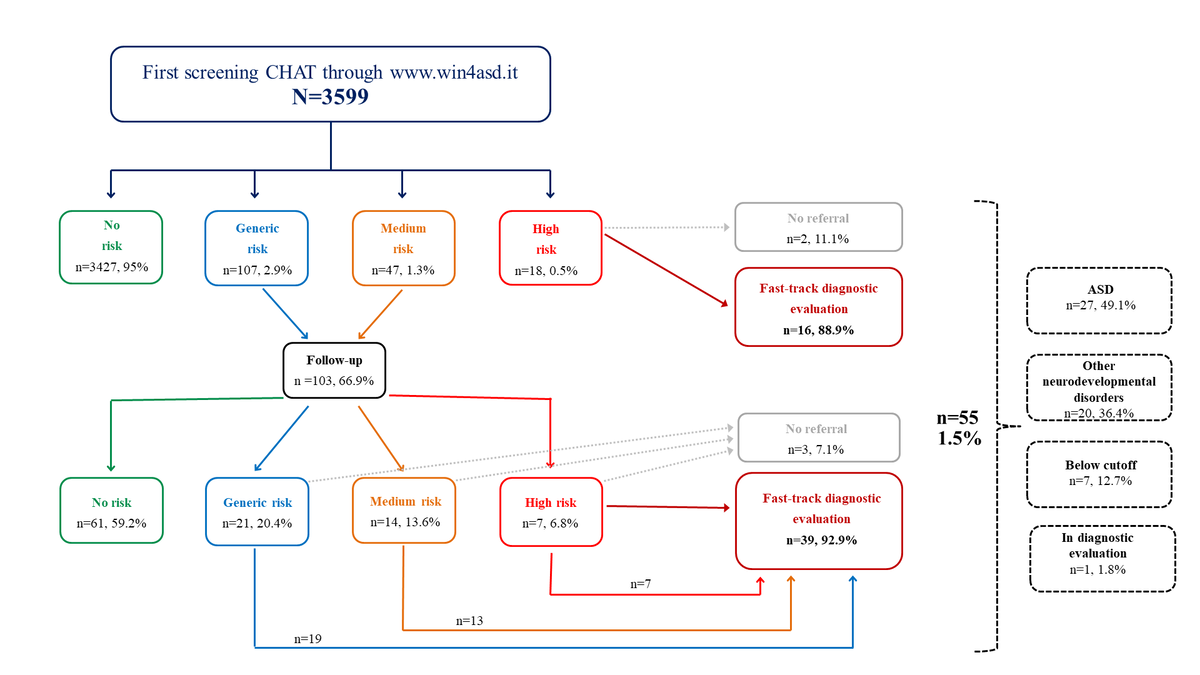
Effectiveness of screening: flowchart of screenings and diagnostic outcomes conducted by family pediatricians from January 2022 to September 2024 in the Valle Olona specialized autism unit subsample. ASD: autism spectrum disorder; CHAT: Checklist for Autism in Toddlers.

The Valle Olona SAU included 62 family pediatricians, of whom 56 (90%) regularly used the screening platform, 2 (3%) had never logged onto it, and 4 (6%) had logged in but did not use it actively. During the study period, 3599 CHAT screenings were performed by primary care pediatricians and specialists. Figure 5 shows the outcomes for patients who completed the CHAT screening at both assessments, including referrals for further diagnostic investigation. In total, 0.1% (2/3599) of the patients were not referred because they had moved away and were no longer followed up on by the SAU. Overall, 1.5% (55/3599) of the total screened children (37/55, 67.3% male; 18/55, 32.7% female) were referred for fast-track diagnostic evaluation at the SAU. Of these 55 children, 27 (49.1%) were diagnosed with ASD and 20 (36.4%) were diagnosed with other neurodevelopmental disorders, including 3 (15%) language disorders (2 male and 1 female), 14 (70%) cases of intellectual disability (n=9, 64.3% male; n=5, 35.7% female), 1 (5%) genetic syndrome (male), and 2 (10%) unspecified neurodevelopmental disorders (1 male and 1 female). In total, 1.8% (1/55) of the children were still awaiting a diagnosis, whereas 12.7% (7/55) exhibited clinical signs but did not meet the diagnostic threshold; in these cases, targeted intervention programs were planned at home and at school, with ongoing 6- and 12-month clinical follow-ups.

Finally, a post hoc power analysis conducted using PASS 2024 (NCSS, LLC) [48] confirmed the adequacy of the Valle Olona SAU subsample (n=3599) to estimate the proportion of children identified as “at risk” (*P*=.01). At a 95% confidence level, the estimated interval ranged from 0.0070 to 0.0138, corresponding to a margin of error of –0.0034 to +0.0034, thus supporting the precision and robustness of the subsample-based estimates.

## Discussion

### Principal Findings

In this work, we presented the Win4ASD platform, an innovative digital tool designed to support early identification and diagnosis of children with ASD within the general population, as a model of preventive medicine. It holds significant potential for building a care network that supports children with ASD from early diagnosis to timely clinical intervention. We described how the platform was integrated into the regional NHS and demonstrated its clinical effectiveness for early ASD screening through a retrospective observational study. The results of this work highlight the political and institutional efforts behind the platform, as well as its clinical impact on the general population.

The first key finding is the commitment of family pediatricians to using the platform owing to the institutional agreement (a supplementary agreement for the Lombardy region) [49]. In total, 72.5% (909/1253) of all family pediatricians of the regional NHS were actively using the platform. This high level of participation makes ASD screening an integral part of the pediatric surveillance system through the systematic use of the CHAT. Furthermore, family pediatricians’ engagement increased over time. We anticipate that continued use of the platform, along with ongoing dissemination and training through institutional channels, will further improve this percentage.

Additionally, our platform enabled the screening of 58,419 toddlers from the general population in Lombardy. According to Italian statistical data (Italian National Institute of Statistics), the estimated pediatric population aged 18 to 22 months between 2022 and 2024 was 69,474 [50]. On the basis of these figures, it can be estimated that 84.08% of children in Lombardy may have received well-child checkups and ASD screening. While a detailed analysis of screening adherence was not possible in this study, a large proportion of families in the region chose to use this free screening service. Moreover, compared with other similar studies that have screened for or diagnosed ASD through digital platforms or telemedicine, our study had a much larger sample size [5,25-27] and is innovative in terms of the network between family pediatricians and local care units for child psychiatry, which the platform succeeded in creating.

On the basis of the total sample (N=58,419), 1% (596/58,419) of the children were classified as “at risk” at the first or repeated screening and were referred for fast-track diagnostic evaluation. Although this proportion is numerically similar to global ASD prevalence estimates, it should be interpreted as the rate of screening positivity, not as a measure of confirmed ASD prevalence [18-20]. The results also show an overall higher number of male than female children. However, the statistically significant comparison between male and female children is probably due to the large sample size, as the absolute differences were quantitatively minimal.

Looking at the data on the effectiveness of screening from a specific SAU, 3599 screenings were performed, and 55 (1.5%) of these children were referred for fast-track diagnostic evaluation. This selection was driven by clinical and organizational considerations and represents a convenience sampling. From further comparative analyses with the general sample and from the post hoc analysis, it can be inferred that all differences between the 2 groups (general and Valle Olona SAU) were quantitatively negligible, supporting the overall homogeneity of the populations, the representativeness of the Valle Olona SAU subsample, and the precision and robustness of the subsample-based estimates.

In this SAU, 90% (56/62) of pediatricians actively used the platform, which led to an adherence rate higher than the regional average, making this SAU an effective model for the screening system. As mentioned above, the data for the total screenings and the percentage of children referred for further diagnosis in this SAU are in line with prevalence data [18-20]. Of the 55 patients referred for evaluation, 45 (81.8%) received a diagnosis, demonstrating the effectiveness of the screening system in identifying clinical signs. Specifically, of the 55 children, 27 (49.1%) received an ASD diagnosis and 20 (36.4%) were diagnosed with other neurodevelopmental disorders. This is in line with the findings of the authors of the CHAT, Baron-Cohen et al [51], who point out that the CHAT intercepts the risk of other neurodevelopmental disorders, especially in the medium or generic risk.

In short, the Win4ASD platform is a viable model for improving the NHS by enhancing continuity of care for children with ASD. Looking ahead, we anticipate even more efficient implementation of the platform, spreading this model further within the NHS and increasing screening coverage across different age groups of the child population. As part of upcoming developments, the Win4ASD platform is being enhanced with the integration of an additional screening tool, the Strengths and Difficulties Questionnaire (SDQ) [52]. The SDQ is a brief behavioral screening questionnaire for children aged 2 to 17 years. It comes in various versions to cater to the needs of researchers, clinicians, and educators. The version for children aged 2 to 4 years is now part of the platform. Similar to the CHAT, the platform allows pediatricians to complete all items of the SDQ, automatically score the screening, and facilitate seamless communication between pediatricians and the SAUs. The SDQ will be available on the platform for children aged 23 to 36 months, providing extended coverage for the active surveillance of neurodevelopmental disorders. The service is free of charge and serves as an additional screening layer within the NHS.

### Limitations

This study has some limitations that must be considered when interpreting its findings. First, this work is not experimental research, which may introduce potential methodological bias. Specifically, we lacked statistical data on the number of children aged 18 to 22 months residing in Lombardy between 2022 and September 2024. Additionally, we did not have data on the number of children in Lombardy diagnosed with ASD or other neurodevelopmental disorders by public or private agencies to compare with the data from the platform.

In addition, we acknowledge that the use of data from a single SAU, along with the lack of information on children at generic, medium, or high risk who, for clinical reasons or parental choice, did not complete the screening and diagnostic process monitored by the platform, represents a limitation regarding the generalizability of our findings. To address this, we plan to expand the outcome dataset by incorporating cases from other SAUs, which will provide a broader and more representative perspective. Nevertheless, despite the potential of the platform, the emerging network, and the high number of screenings carried out, our results cannot yet be generalized to the wider regional context.

Furthermore, as illustrated in the flowcharts (Figures 4 and 5), some children identified as being at risk of neurodevelopmental disorders through the CHAT were not referred to SAU services for diagnostic assessment. This may be due to a variety of clinical and nonclinical factors, for instance, parental decisions regarding the continuation of diagnostic evaluation or a family’s relocation to another region.

### Conclusions

Owing to the political and institutional support that the platform has received over the years, it has been possible to make improvements that have led to an increasingly widespread dissemination of the screening system. Early detection of risk and timely intervention can prevent the progressive development of behavioral atypicalities; have a significant impact on the quality of life of the child and their family; and, finally, allow for a reduction in health care costs and a consequent improvement in the organization of the health care system.

## Abbreviations

ADI-R: Autism Diagnostic Interview–Revised
ADOS-2: Autism Diagnostic Observation Schedule, Second Edition
ASD: autism spectrum disorderCHAT: Checklist for Autism in ToddlersNHS: National Health Service
SAU: specialized autism unit
SDQ: Strengths and Difficulties Questionnaire
Win4ASD: Web Italian Network for Autism Spectrum Disorder
