# Correction: Rising to the Challenge of Early Screening in Primary Health Care Through the Web Italian Network for Autism Spectrum Disorder (Win4ASD) in the Pediatric Population: Retrospective Observational Study

**DOI:** 10.2196/106387

**Published:** 2026-07-15

**Authors:** Noemi Buo, Eleonora Rosi, Silvia Busti Ceccarelli, Mariarosa Ferrario, Valerio Maiorca, Erika Morandi, Nicole Viganó, Ivan Limosani, Laura Falcone, Massimo Molteni, Paola Colombo

**Affiliations:** 1 Child Psychopathology Unit-Smart Lab Scientific Institute, Istituto di Ricovero e Cura a Carattere Scientifico (IRCCS) Eugenio Medea Bosisio Parini, Lecco Italy; 2 Azienda Socio Sanitaria Territoriale della Valle Olona - Dipartimento di salute mentale e delle dipendenze Gallarate, Varese Italy; 3 Direzione Generale Welfare, Lombardy Region Milan Italy

In “Rising to the Challenge of Early Screening in Primary Health Care Through the Web Italian Network for Autism Spectrum Disorder (Win4ASD) in the Pediatric Population: Retrospective Observational Study” [1], the authors noted one error.

The following affiliation has been revised:

Child Psychopathology Unit-Smart Lab, Scientific Institute, Istituto di Ricovero e Cura a Carattere Scientifico (IRCCS) Eugenio Medea, Bosisio Parini, Lecco, Lazio, Italy

After removing “Lazio,” the affiliation now reads:

Child Psychopathology Unit-Smart Lab, Scientific Institute, Istituto di Ricovero e Cura a Carattere Scientifico (IRCCS) Eugenio Medea, Bosisio Parini, Lecco, Italy

The correction will appear in the online version of the paper on the JMIR Publications website, together with the publication of this correction notice. Because this was made after submission to PubMed, PubMed Central, and other full-text repositories, the corrected article has also been resubmitted to those repositories.
